# Novel Multi-Segment Foot Model Incorporating Plantar Aponeurosis for Detailed Kinematic and Kinetic Analyses of the Foot With Application to Gait Studies

**DOI:** 10.3389/fbioe.2022.894731

**Published:** 2022-06-24

**Authors:** Yuka Matsumoto, Naomichi Ogihara, Hiroki Hanawa, Takanori Kokubun, Naohiko Kanemura

**Affiliations:** ^1^ Graduate School of Saitama Prefectural University, Graduate Course of Health and Social Services, Saitama, Japan; ^2^ Department of Biological Sciences, The University of Tokyo, Tokyo, Japan; ^3^ Department of Health Science, University of Human Arts and Sciences, Saitama, Japan; ^4^ Department of Health and Social Services, Saitama Prefectural University, Saitama, Japan

**Keywords:** multi-segment foot model, inverse dynamics, plantar fascia, walking, healthy adults, motion analysis, foot kinematics, foot kinetics

## Abstract

Kinetic multi-segment foot models have been proposed to evaluate the forces and moments generated in the foot during walking based on inverse dynamics calculations. However, these models did not consider the plantar aponeurosis (PA) despite its potential importance in generation of the ground reaction forces and storage and release of mechanical energy. This study aimed to develop a novel multi-segment foot model incorporating the PA to better elucidate foot kinetics. The foot model comprised three segments: the phalanx, forefoot, and hindfoot. The PA was modeled using five linear springs connecting the origins and the insertions *via* intermediate points. To demonstrate the efficacy of the foot model, an inverse dynamic analysis of human gait was performed and how the inclusion of the PA model altered the estimated joint moments was examined. Ten healthy men walked along a walkway with two force plates placed in series close together. The attempts in which the participant placed his fore- and hindfoot on the front and rear force plates, respectively, were selected for inverse dynamic analysis. The stiffness and the natural length of each PA spring remain largely uncertain. Therefore, a sensitivity analysis was conducted to evaluate how the estimated joint moments were altered by the changes in the two parameters within a range reported by previous studies. The present model incorporating the PA predicted that 13%–45% of plantarflexion in the metatarsophalangeal (MTP) joint and 8%–29% of plantarflexion in the midtarsal joints were generated by the PA at the time of push-off during walking. The midtarsal joint generated positive work, whereas the MTP joint generated negative work in the late stance phase. The positive and negative work done by the two joints decreased, indicating that the PA contributed towards transfer of the energy absorbed at the MTP joint to generate positive work at the midtarsal joint during walking. Although validation is limited due to the difficulty associated with direct measurement of the PA force *in vivo*, the proposed novel foot model may serve as a useful tool to clarify the function and mechanical effects of the PA and the foot during dynamic movements.

## 1 Introduction

The multi-segment foot model, such as the Oxford foot model (Carson et al., 2001; Wright et al., 2011; Li et al., 2022) and the Leardini foot model (Leardini et al., 2007; Deschamps et al., 2012; Watari et al., 2021), was developed to replace the conventional single-segment foot model for detailed *in vivo* evaluation of the foot segment kinematics during movements. Its clinical utility has been highly appreciated following assertions by several studies that foot kinematics are affected by age (Arnold et al., 2014; Deschamps et al., 2017), sex (Takabayashi et al., 2018; Sekiguchi et al., 2020), and deformities such as flat foot (Kim et al., 2020) or hallux valgus (Shin et al., 2019). However, detailed kinetics of the human foot during dynamic movements have not been similarly investigated, mainly due to the unavailability of a detailed foot model to estimate internal forces and moments within the foot segments. Kinetic assessments of the human foot during dynamic movements are particularly important since larger forces are applied to the foot and movements of the foot bones are greater in dynamic movements, possibly associated to the pathogenesis of foot injuries or disorders.

Recently, a few kinetic multi-segment foot models have been used to calculate the inter-foot segment moments based on an inverse dynamics analysis during gait (Bruening et al., 2012; Dixon et al., 2012; Saraswat et al., 2014; Kevin et al., 2017). However, these models lacked anatomical accuracy for two main reasons: 1) they did not calculate the detailed inertial properties of the divided foot, and 2) they did not consider forces generated by the plantar aponeurosis (PA). In previous studies, the mass and inertial tensor of each foot segment were determined arbitrarily (Dixon et al., 2012) or calculated by assuming a mathematical model such as a cylinder (Bruening et al., 2012; Saraswat et al., 2014; Kevin et al., 2017); however, such an assumption is unreasonable as the radius and height of each foot segment are not uniform. Although the influence of the inertia of the foot is expected to be relatively minor due to the small mass of the foot (Zelik and Honert, 2018), the acceleration in the most distal segment of the leg could be large, particularly in dynamic movements such as running and jumping. Therefore, accurate identification of the inertial parameters of each foot segment might be important for inverse dynamics analysis to calculate the joint moment of the foot.

The PA is an elastic band that supports the longitudinal foot arch, which consists of high density of collagen fibers. The occurrence of the windlass mechanism at push-off during walking and running has been proposed, wherein dorsiflexion of the metatarsophalangeal (MTP) joint winds the PA around the metatarsal heads, thereby increasing the rigidity of the foot (Hicks, 1954). Based on an inverse kinematic analysis, a study reported that the PA generates a tension force that is 1.5 times the bodyweight (Caravaggi et al., 2009), indicating that the forces generated by the PA are quite large, potentially having a major effect on the joint moments and forces computationally estimated based on an inverse dynamics analysis. Further, wire electromyographic analysis has shown that both the PA and the plantar intrinsic foot muscles contribute to increasing the foot rigidity in response to the magnitude of forces, such as stronger push-off during walking or running (Péter et al., 2015; Kelly et al., 2019; Farris et al., 2020). However, measuring the electromyography of the intrinsic muscles of the foot is difficult due to the invasive methods required. Dynamic finite element models of the human foot (e.g., Ito et al., 2022) can be used to estimate forces and moments generated by the foot muscles, but they are of limited utility due to large computational cost. Therefore, to evaluate foot function and elucidate the pathogenesis of foot injuries or disorders, it would be useful to establish a multi-segment kinetics model of the foot incorporating the PA that can be applied non-invasively during locomotion to estimate more accurate forces and moments in the foot.

In this study, we aimed to develop a novel multi-segment foot model to analyze foot kinematics and kinetics during dynamic movements by incorporating more accurate inertial parameters and the PA, and to demonstrate the efficacy of the developed foot model by applying it to gait analysis. Efforts have been previously made to incorporate the PA in a biomechanical foot model to estimate the mechanical contribution of the PA (Caravaggi et al., 2009; Chen et al., 2019; Farris et al., 2020; Welte et al., 2021). However, these studies estimated only the forces generated by the PA, but not the resultant joint moments within the foot segments generated during movements. For this purpose, a multi-segment foot model is necessary, but no studies have attempted to incorporate the PA in a multi-segment foot model for estimation of the resultant joint moments that can otherwise only be quantified using invasive techniques.

## 2 Materials and Methods

### 2.1 Model

**FIGURE 1:**
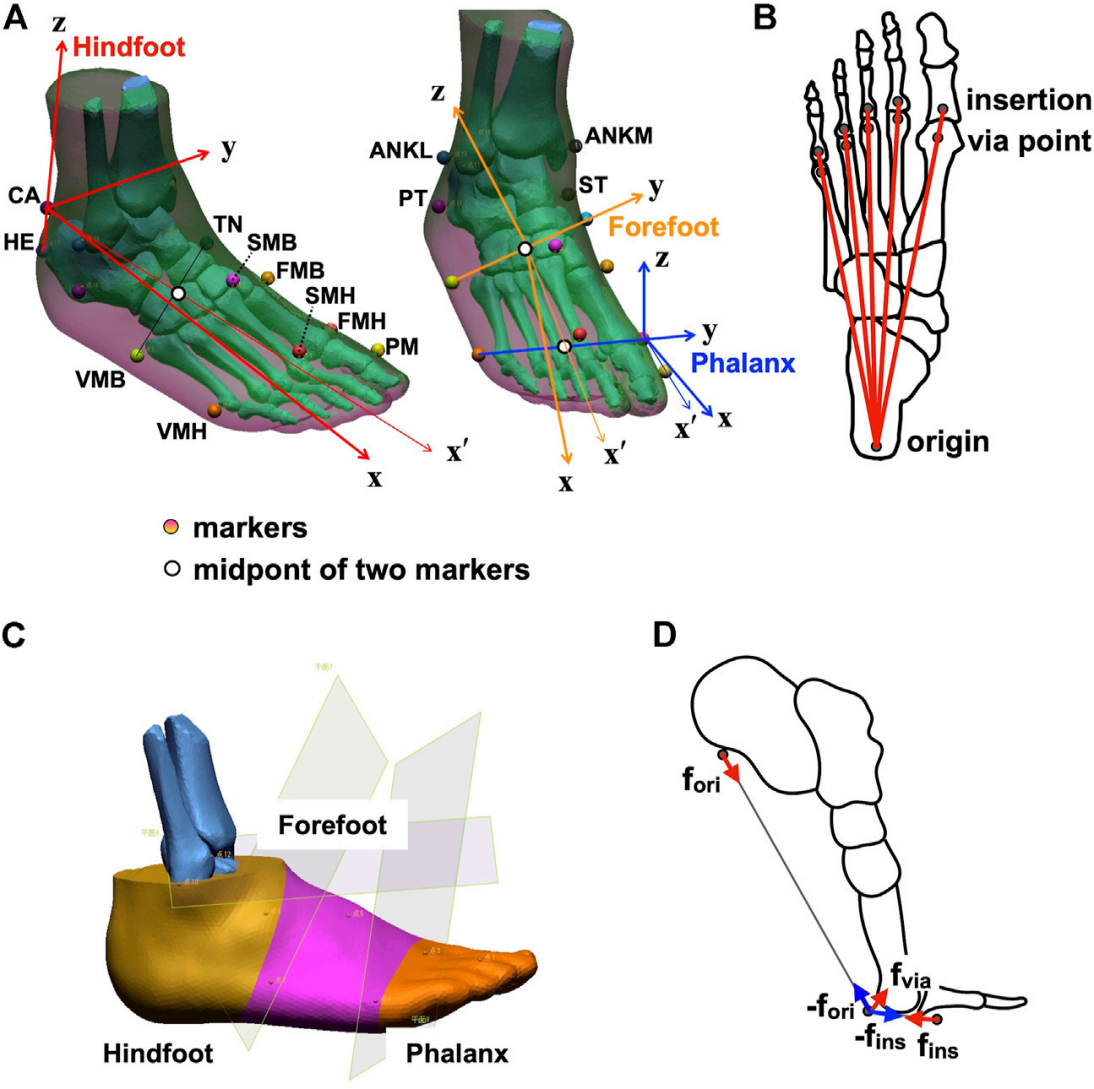
Multi-segment foot model incorporating the plantar aponeurosis (PA) for detailed kinematic and kinetic analyses of the foot. **(A)** Marker placements and the definitions of the coordinate systems of the phalanx, forefoot, and hindfoot segments. **(B)** The model of PA. The PA was modeled as five linear springs connecting the origin, *via* point, and insertion. **(C)** Segmentation of the phalanx, forefoot, and hindfoot segments. **(D)** Forces applied to the origin, *via* point, and insertions of the PA.

To develop an anatomically accurate multi-segment foot model, computed tomography (CT) data of the foot of an adult male (age: 42 years, weight: 72 kg, height: 172 cm) were obtained (Ito et al., 2022). Three-dimensional surface models of the foot surface and skeleton were constructed (Figure 1A) using segmentation software (Analyze 9.0, Biomedical Imaging Resource, Mayo Clinic, Rochester, MN, United States). The human foot was represented as a chain comprising three segments (phalanx, forefoot, and hindfoot segments) based on the marker positions attached to anatomical landmarks that are widely used in kinematic studies (Leardini et al., 2007) (Table 1; Figure 1A). The joints between the tibia and hindfoot (ankle joint), hindfoot and forefoot (midtarsal joint), and forefoot and phalanx segments (MTP joint) were defined as the midpoints between the lateral and medial malleoli (ANKL and ANKM), the navicular tubercle (TN) and the fifth metatarsal base (VMB), and the heads of the first and fifth metatarsals (FMH and VMH), respectively. The coordinate systems of the three segments were defined as follows: the *y* and *z* axes of the hindfoot segment were defined as the normal vector of the plane defined by the superior (CA) and inferior (HE) points of the calcaneal tuberosity and the midtarsal joint, and the axis connecting the CA and HE, respectively; those of the forefoot segment were defined as the axis connecting the TN and VMB, and the normal vector of the plane defined by the TN, VMB, and MTP joints, respectively; those of the phalanx segment were defined as the axis connecting the FMH and VMH, and the normal vector of the plane defined by FMH, VMH, and the head of the first proximal phalanx (PM), respectively.

**TABLE 1:** Definition of marker placement.

Name	Description
PM	Dorso-medial aspect of the first proximal phalanx head
FMH	Dorso-medial aspect of the first metatarsal head
SMH	Dorso-medial aspect of the second metatarsal head
VMH	Dorso-lateral aspect of the fifth metatarsal head
FMB	Dorso-medial aspect of the first metatarsal base
SMB	Dorso-medial aspect of the second metatarsal base
VMB	Dorso-lateral aspect of the fifth metatarsal base
TN	Most medial apex of the navicular bone
ST	Most medial apex of the sustentaculum tali
PT	Lateral apex of the peroneal tubercle
CA	Superior apex of the calcaneus
HE	Apex of the calcaneal tuberosity
ANKL	Distal apex of the lateral malleolus
ANKM	Distal apex of the medial malleolus

We represented the PA using five linear springs (PA1−5, from medial to lateral) connecting the hindfoot and phalanx segments *via* a point on the metatarsal plantar surface (Figure 1B). The positions of the origin, *via* point, and insertion of the PA were obtained from the CT data of the foot (Ito et al., 2022). The origin of the PA was defined as the plantar surface of the calcaneal tuberosity. The *via* points were defined as the plantar surfaces of the sesamoid of the first metatarsal and the second to fifth metatarsal heads. The insertion was defined as the plantar surface of the base of the first to fifth proximal phalanges. The *via* points were necessary to model the wrapping paths of PA slips around the metatarsal heads, analogous to the pully model in Caravaggi et al. (2009). To create a subject-specific model, the positions of the origin, *via* point, and insertion extracted from the CT data were described in the segment coordinate systems of the hindfoot, forefoot, and phalanx segments, the origins of which were HE, VMB, and VMH, respectively, and scaled by the length of the line connecting ANKL and VMH.

The length of the *i*th PA was calculated as the sum of the lengths from the origin to the *via* point and from the *via* point to insertion as:

$$L_{i}=\left|p_{ins\_i}-p_{via\_i}\right|+\left|p_{via\_i}-p_{ori\_i}\right|\;$$ (1)

The PA tension force ( $f_{PAi}$ ) can be calculated as:

$$f_{PAi}=\max\left(0,\;k_{i}\left(L_{i}-L_{0i}\right)\right)$$ (2)

where $k_{i}$ is the spring constant and $L_{0i}\;$ is the natural length of the *i*th PA. The natural length of the PA was estimated to be 0.98 times the PA length during quiet standing ( $L_{qs\_i}$ ), since the foot (hence, the PA) should be slightly stretched from its natural state due to flattening of the foot arch during quiet standing. The spring constants of the PAs were calculated based on studies reporting that the strain (ε) of the PA was approximately 0.07 (the range of 0.03–0.12 based on Caravaggi et al. (2009, 2010) and Gefen (2003), and the force generated by the PA was approximately 1.5 times the body weight (Caravaggi et al., 2009) at the time of push-off during walking. The spring constant can be calculated as:

$$k_{i}=\frac{\left(9.8\right)\left(1.5BW\right)}{5\left(0.07\right)\left(0.98L_{qs\_i}\right)}$$ (3)

where BW is the individual-specific body weight. Therefore, the spring constant varies by individual depending on the body weight, but this is reasonable since heavier individuals have thicker (Pascual Huerta and Alarcón García, 2007) and hence harder PAs (the spring constant is proportional to the cross-sectional area of the PA if the material property is the same; see Ito et al., 2022). The tension force vectors ( $f_{ins}^{PAi}$ , $f_{ori}^{PAi}$ , $f_{via}^{PAi}$ ) acting on the insertion, origin, and *via* point of the PA can be defined as:

$$f_{ins}^{PAi}=f_{PAi}\frac{p_{via\_i}-p_{ins\_i}}{\left|p_{via\_i}-p_{ins\_i}\right|}\;$$ (4)

$$f_{ori}^{PAi}=f_{PAi}\frac{p_{via\_i}-p_{ori\_i}}{\left|p_{via\_i}-p_{ori\_i}\right|}\;$$ (5)

$$f_{via}^{PAi}=\left(-f_{ins}^{PAi}\right)+\left(-f_{ori}^{PAi}\right)$$ (6)

**FIGURE 2:**
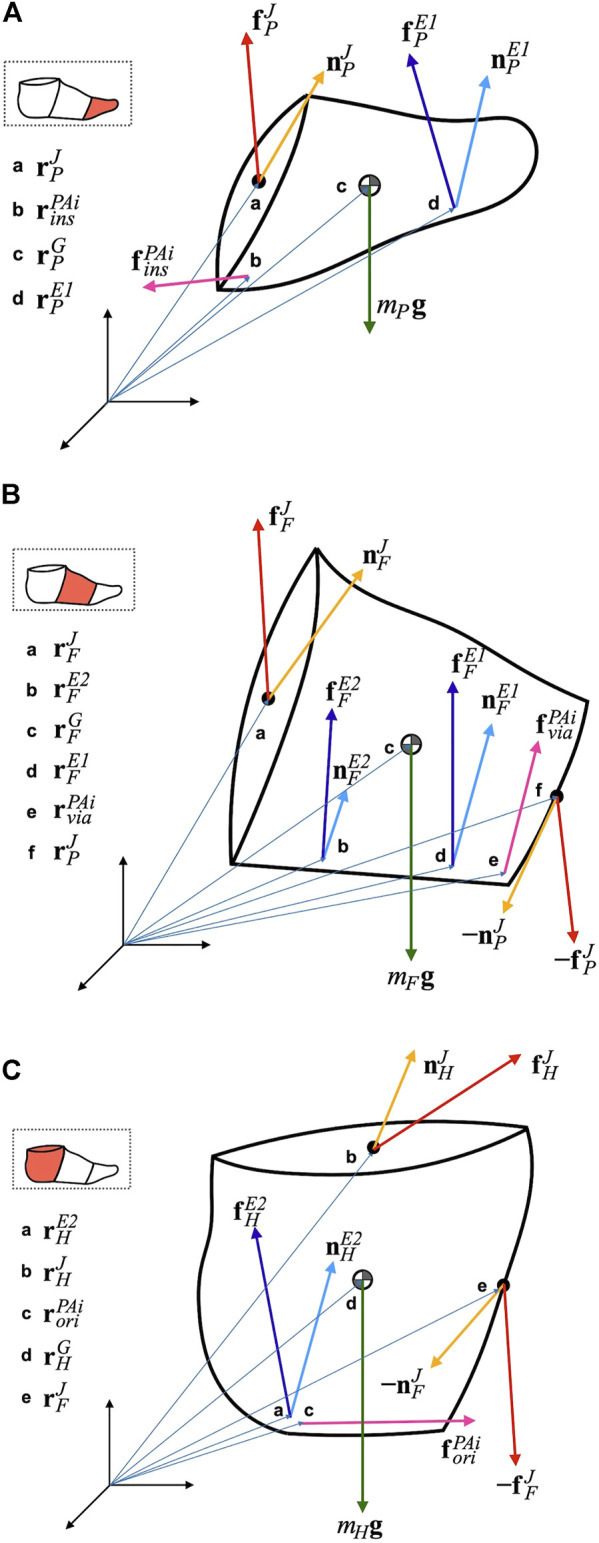
Free body diagram of the phalanx **(A)**, forefoot **(B)**, and hindfoot segment **(C)**. See Table 2 for notations.

Based on the free body diagrams in Figure 2, the Newton−Euler equations of the motion of the multi-segment foot model consisting of the phalanx, forefoot, and hindfoot segments can be written as:

$$m_{P}{\ddot{r}}_{P}^{G}=f_{P}^{J}+f_{P}^{E1}+\overset{5}{\underset{i=1}{\sum }}f_{ins}^{PAi}+m_{P}g$$ (7)

MP{ωP×(IPPω)+IPPω˙}=nPJ+(rPJ−rPG)×fPJ+(rPE1−rPG)×fPE1+nPE1+∑i=15(rinsPAi−rPG)×finsPAi (8)

$$m_{F}{\ddot{r}}_{F}^{G}=f_{F}^{J}+\left(-f_{P}^{J}\right)+f_{F}^{E1}+f_{F}^{E2}+\overset{5}{\underset{i=1}{\sum }}f_{via}^{PAi}+m_{F}g$$ (9)

MF{ωF×(IFFω)F+IFω˙}=nFJ+(−nPJ)+(rFJ−rFG)×fFJ+(rPJ−rFG)×(−fPJ)+(rFE1−rFG)×fFE1+nFE1+(rFE2−rFG)×fFE2+nFE2+∑i=15(rvia PAi−rFG)×fvia PAi (10)

$$m_{H}{\ddot{r}}_{H}^{G}=f_{H}^{J}+\left(-f_{F}^{J}\right)+f_{H}^{E2}+\overset{5}{\underset{i=1}{\sum }}f_{ori}^{PAi}+m_{H}g$$ (11)

MH{ωH×(IHHω)+IHHω˙}=nHJ+(−nFJ)+(rHJ−rHG)×fHJ+(rFJ−rHG)×(−fFJ)+(rHE2−rHG)×fHE2+nHE2+∑i=15(roriPAi−rHG)×foriPAi (12)

The variables in the equations are presented in Table 2. The left superscripts *P*, *F* and *H* denote the coordinate systems in which the corresponding vector or matrix was represented.

**TABLE 2:** Notations in the equations of motion.

i	segment or joint ID. P, F, and H represents phalanx, forefoot, and hindfoot segment, respectively. The *i*th joint is the proximal joint of the *i*th *segment*.
$m_{i}$	mass of *segment i*
$r_{i}^{G}$	position vector of the center of mass of *segment i*
g	gravitational acceleration vector
$M_{i}$	orthonormal basis matrix of the segment coordinate system of *segment i*
$I_{i}$	inertial tensor around the center of mass of *segment i*
$\omega_{i}$	angular velocity vector of *segment i*
$r_{i}^{J}$	position vector of *joint i*
$r_{i}^{E1}$	position vector of the center of pressure on the force plate 1 acting on *segment i*
$r_{i}^{E2}$	position vector of the center of pressure on the force plate 2 acting on *segment i*
$r_{ori}^{PAi}$	position vector of the origin of the *i*th PA
$r_{via}^{PAi}$	position vector of the *via* point of the *i*th PA
$r_{ins}^{PAi}$	position vector of the insertion of the *i*th PA
$f_{i}^{J}$	joint reaction force vector of *joint i*
$n_{i}^{J}$	joint moment vector of *joint i*
$f_{i}^{E1}$	ground reaction force of the force plate 1 acting on *segment i*.
$n_{i}^{E1}$	ground reaction moment of the force plate 1 acting on *segment i*
$f_{i}^{E2}$	ground reaction force of the force plate 2 acting on *segment i*.
$n_{i}^{E2}$	ground reaction moment of the force plate 2 acting on *segment i*
$f_{ori}^{PAi}$	tension force acting on the origin of the *i*th PA
$f_{via}^{PAi}$	tension force acting on the *via* point of the *i*th PA
$f_{ins}^{PAi}$	tension force acting on the insertion of the *i*th PA

### 2.2 Inertial Properties

To calculate the inertial properties (i.e., mass, inertial tensor around the center of mass (COM), and position of the center of mass in each segment), the foot surface was divided into segments by planes passing through ANKL and ANKM, TN and VMB, and FMH and VMH (Figure 1C). Therefore, the hindfoot, forefoot, and phalanx segments correspond to the calcaneus, talus, cuboid, and navicular; cuneiforms and metatarsals; and five phalanges, respectively. The inertial parameters of each segment were calculated using a computer-aided design software (Autodesk Inventor Professional, Autodesk, United States), assuming a homogeneous segment composition and a density of 1.1 g/cm^3^ (Winter, 1990). To create an individual-specific foot model, the relative mass of the foot segments and the relative inertial tensor around the segment COM were computed based on the inertial parameters from the CT data. Relative foot segment mass was defined as the mass of the foot segment as a percentage of the total foot mass. The relative inertial tensor around the segment COM was defined as the inertial tensor around the segment COM normalized by 5/3 power of each segment mass. Relative COM position was defined as the location of the COM, expressed as a percentage of the segment length from the proximal point of the segment, assuming that the COM is located on the line connecting the centers of the proximal and distal joints.

### 2.3 Participants

Ten males (age: 23.9 ± 3.0 years, height: 171.8 ± 5.1 cm, weight: 62.8 ±8.2 kg) without any deformity of the foot and lower extremity and with no history of orthopedic, neurological, and musculoskeletal disorders that are likely to affect gait were recruited for data collection. The number of participants was determined by referring to previous studies (Leardini et al., 2007; Portinaro et al., 2014). All participants provided written informed consent following a detailed explanation of the study’s purpose and the risks involved. The experimental procedures used in this study complied with the Declaration of Helsinki and were approved by the Ethics Committee on Human Experimentation at Saitama Prefectural University (No. 29508).

### 2.4 Experimental Procedure

Infrared-reflecting markers (diameters: 9.5 and 14 mm) were attached to 65 landmarks on the foot and the whole body, according to Table 1 and Figure 1A, and the Plug-in-Gait Full-body Ai model (Davis et al., 1991; Vicon Motion Systems Limited, 2016) (Supplementary Figure S1; Supplementary Table S1), respectively. The participants walked at a self-selected speed along a walkway with four force plates (two per side) placed in series close together. The three attempts in which the fore- and hindfoot contacted the front and rear force plates, respectively (Supplementary Figure S2), were selected for the inverse dynamic analysis.

Marker trajectories were collected using the Vicon Nexus 2.10.2, a three-dimensional motion analysis system (Vicon, Oxford, United Kingdom) with 20 infrared cameras at 100 Hz. Ground reaction force was collected from four force plates (Kistler Instrumente AG, Winterthur, Switzerland) at 1,000 Hz. All data were synchronized using the Vicon Workstation v4.5 software and saved for offline analysis.

### 2.5 Data Processing and Analysis

Marker trajectories and ground reaction forces were filtered using a zero-phased lag and fourth-order Butterworth filter with a cutoff frequency of 10 Hz. The 3D rotation angles of the ankle joint were described by the y-x-z Euler angle, and those of the midtarsal and MTP joints were described by z-x-y Euler angles. The rotational angles around the y-, x-, z-axes represent plantarflexion–dorsiflexion, inversion–eversion and adduction−abduction, respectively. The MTP joint in the present model is regarded as a hinge joint allowing only plantarflexion–dorsiflexion; hence the rotational angles around the other two axes are not presented.

For inverse dynamics, the total mass of each individual’s foot was estimated to be 0.0145 times the body weight of the individual (Winter, 1990). The mass, inertial tensor, and COM position of each foot segment were then calculated based on the relative mass, inertial tensor, and COM position obtained from the CT scan of the foot used to create the present multi-segment foot model (See Section 2.1). The ground reaction forces were applied to the foot segments based on the force plate data. The positional relationship between the center of pressure and the markers specified the segments on which the two force vectors were applied. The tension forces due to the PAs were applied to the insertions, *via* points, and origins of the PAs (Figure 1D). The joint moments were calculated by solving the equations for motions (Eqs 7–12) consecutively from the distal phalanx segment to the proximal hindfoot segment. The calculated joint moment vectors were transformed to the corresponding proximal segment coordinate systems to match them with the joint angles. The joint powers were calculated by multiplying the joint angular velocities and joint moments. The joint power curves were integrated into the positive and negative portions during one gait cycle, which represented energy generation and energy absorption, respectively. All data were analyzed using MATLAB 2018a (MathWorks, Natick, MA, United States).

In this study, the inverse dynamic analysis was performed with and without the PA to investigate how inclusion of the PA in the model alters the estimated joint moments and work. For this, the maximum and minimum values of the calculated joint moments during one gait cycle were compared between the two conditions. In addition, the PA contribution rate (%PA contribution) was calculated as the percentage of the joint moment generated by the PA with respect to the net joint moment, to quantify the contribution of the PA on joint moment generation at the time of push-off during walking. The changes in the positive and negative work generated or absorbed during walking were also quantified and compared.

### 2.6 Sensitivity Analysis

We estimated the relative mass of the segment and the inertia tensor of each segment from the CT data of a single male participant. Further, due to the uncertainty regarding the stiffness and the natural length of each PA spring, a sensitivity analysis was conducted to evaluate how the estimated joint moments were altered by the changes in these parameters within a reasonable range. For the mass and inertia of the segment, we made 0.5- and 1.5-fold changes in the mass and inertial parameters of the foot model to assess how changes in the mass and inertial parameters affected the joint moments, which was calculated using inverse dynamics. We made the 0.5- and 1.5-fold changes in the mass and inertial parameters because the standard deviations of the mass and inertial parameters were reportedly about a quarter of the respective mean values (Zatsiorsky, 2002) and approximately 95% of the population lies within two standard deviations. For the stiffness, we decreased and increased the natural PA length (default $L_{0i}=0.98L_{qs\_i}$ ) by $0.02L_{qs\_i}$ , and the PA strain at toe-off (default ε = 0.07) by 0.03 to assess how changes in the stiffness altered the results. The ranges were determined by referring to previous studies (Gefen, 2003; Caravaggi et al., 2009).

### 2.7 Statistical Analysis

To test for possible statistical differences in the joint moment and power profiles between the models with and without PA in a continuous manner, we performed a one-dimensional Statistical Parametric Mapping (SPM) paired *t*-test (Pataky, 2012). To compare the statistical differences in the estimated peak joint moments, joint works, and %PA contributions, we performed a paired *t*-test if the normality was confirmed using the Kolmogorov–Smirnov test. If the normality was violated, we used a signed-rank test for statistical comparisons. All statistical tests were performed with a significance level of 5% using MATLAB 2018a (MathWorks, Natick, MA, United States).

## 3 Results

### 3.1 Inertial Properties of the Foot

The relative mass of the segment, relative COM position, and relative inertial tensor around the COM of the phalanx, forefoot, and hindfoot segments are shown in Table 3. The size-normalized position vectors of the insertions, *via* points, and origins of the PAs are presented in Table 4. The mean and standard deviations of the masses of the phalanx, forefoot, and hindfoot segments of the 10 participants were calculated to be 0.131 ± 0.017 kg, 0.386 ± 0.051 kg, and 0.393 ± 0.052 kg, respectively.

**TABLE 3:** Relative segment mass, relative center of mass position, and relative inertial tensor around the center of mass of each phalanx, forefoot, and hindfoot segment.

Foot segment	Relative foot segment mass, %	Relative COM position, %	Relative inertial tensor around the COM, arbitrary unit					
			Ixx	Iyx	Iyy	Izx	Izy	Izz
Phalanx	14.4	43.6	2.55 × 10^−3^	−0.428 × 10^−3^	1.43 × 10^−3^	−0.220 × 10^−3^	−0.0507 × 10^−3^	3.38 × 10^−3^
Forefoot	42.4	41.9	1.40 × 10^−3^	−0.000748 × 10^−3^	1.73 × 10^−3^	−0.0510 × 10^−3^	−0.117 × 10^−3^	2.20 × 10^−3^
Hindfoot	43.2	55.4	1.54 × 10^−3^	0.00986 × 10^−3^	1.84 × 10^−3^	−0.195 × 10^−3^	−0.155 × 10^−3^	1.48 × 10^−3^

**TABLE 4:** Size-normalized position vectors of the insertions, *via* points, and origins of the plantar aponeuroses.

	ins_1	ins_2	ins_3	ins_4	ins_5	*via*_1	*via*_2	*via*_3	*via*_4	*via*_5	origin
*x*	0.110	0.173	0.174	0.123	0.084	0.660	0.692	0.642	0.578	0.496	0.284
*y*	0.689	0.510	0.396	0.291	0.158	0.427	0.336	0.244	0.136	0.032	0.051
*z*	−0.080	−0.043	−0.054	−0.061	−0.062	−0.192	−0.059	−0.064	−0.069	−0.054	−0.107

### 3.2 Foot Kinematics

**FIGURE 3:**
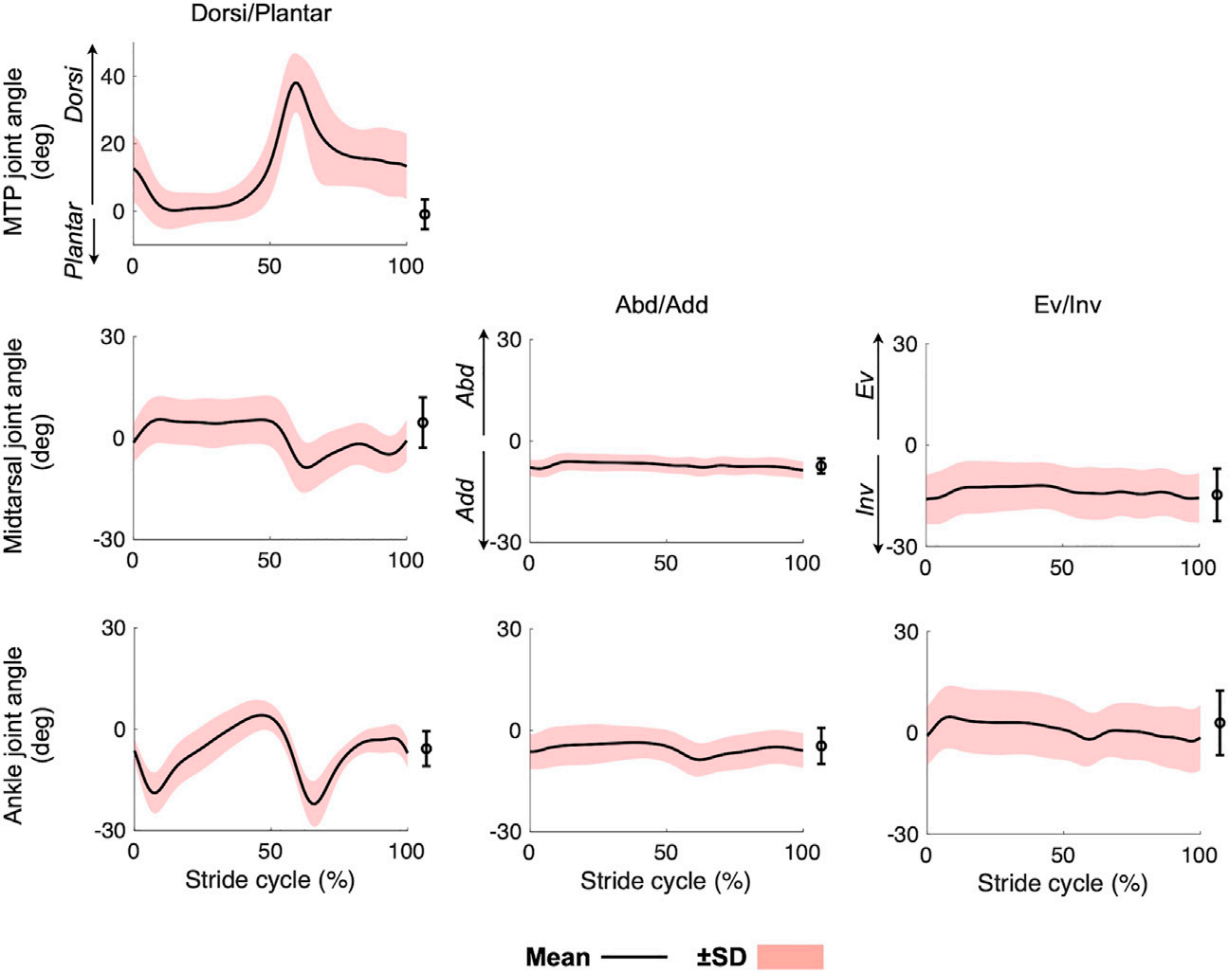
Mean joint angle profiles of the foot during walking. Mean (solid line) ± standard deviation (red band). Means and standard deviations of the joint angles during quiet standing were also plotted on the right side of each graph.

The mean and standard deviations of the speed, cycle duration, and stance phase duration of the measured walking were 1.33 ± 0.17 m/s, 1.03 ± 0.05 s and 0.63 ± 0.05 s, respectively. The 3D joint angle profiles of the MTP, midtarsal, and ankle joints during walking are presented in Figure 3. The joint angles during quiet standing are also presented as a reference point. The joints angles were positive for eversion, dorsiflexion, and abduction. The ankle joint plantarflexed after heel-contact and dorsiflexed during single support phase so that the shank vaulted over the foot. The joint then plantarflexed while approaching toe-off and returned to its natural angle during the swing phase. Following heel contact, the midtarsal and MTP joints dorsiflexed and plantarflexed, respectively. During the single support phase, both joints remained largely unchanged; however, in the late stance phase, the midtarsal and MTP joints plantarflexed and dorsiflexed, respectively. Eversion−inversion and abduction−adduction of the two joints were relatively small, though slight eversion of the ankle and midtarsal joints and abduction of the ankle joint were observed during the stance phase.

**FIGURE 4:**
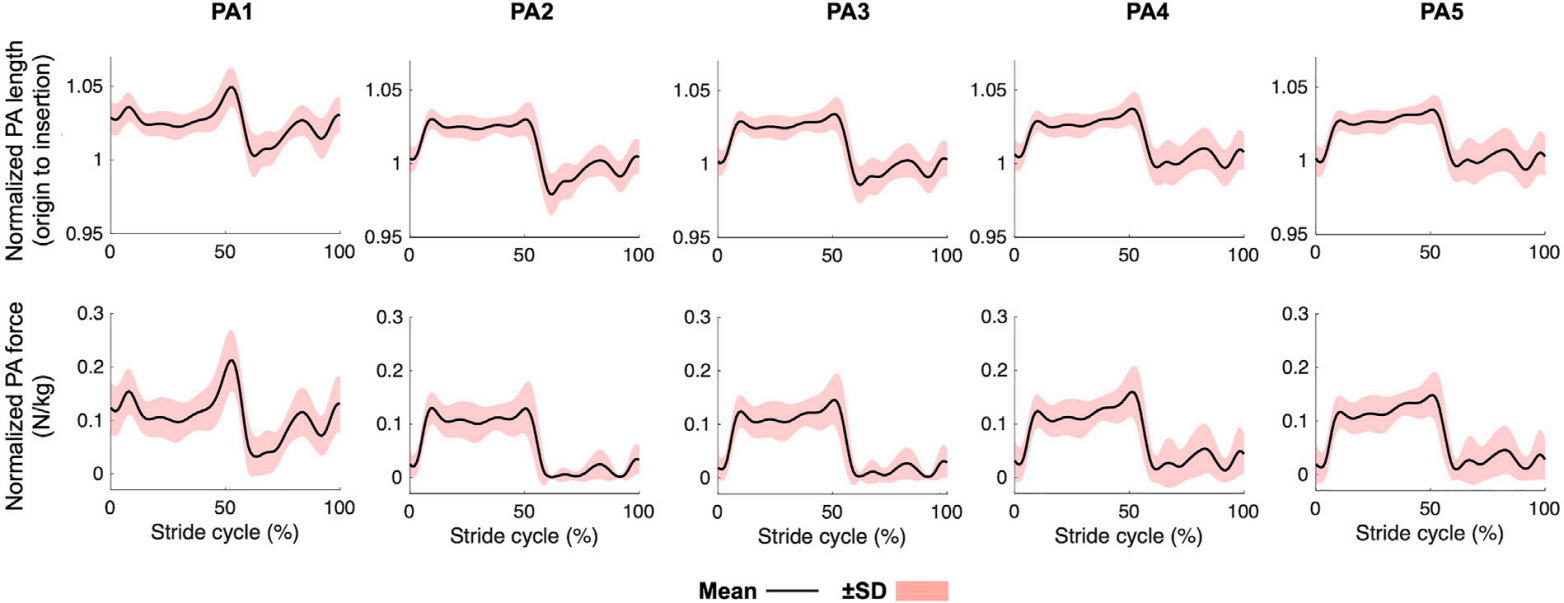
Mean plantar aponeurosis length and force profiles during walking. Mean (solid line) ± standard deviation (red band).

Figure 4 displays the changes in the PA length and force during walking. The length profiles were generally consistent with each other for the four lateral PAs. They were stretched after the heel-contact and remained stretched until 50% of the gait cycle. During push-off, they were sharply shortened and remained shortened during the swing phase. PA1 was more largely stretched at 50% of the gait cycle than the other four PAs, mainly due to the elongation of the distal portion of the PA connecting the *via* point and insertion. The maximum tension forces exerted by the five PAs were 0.23 ± 0.06 N/kg, 0.15 ± 0.04 N/kg, 0.16 ± 0.04 N/kg, 0.17 ± 0.04 N/kg, 0.16 ± 0.04 N/kg, respectively. The resultant maximum net PA force was 0.87 ± 0.20 N/kg.

### 3.3 Foot Kinetics

**FIGURE 5:**
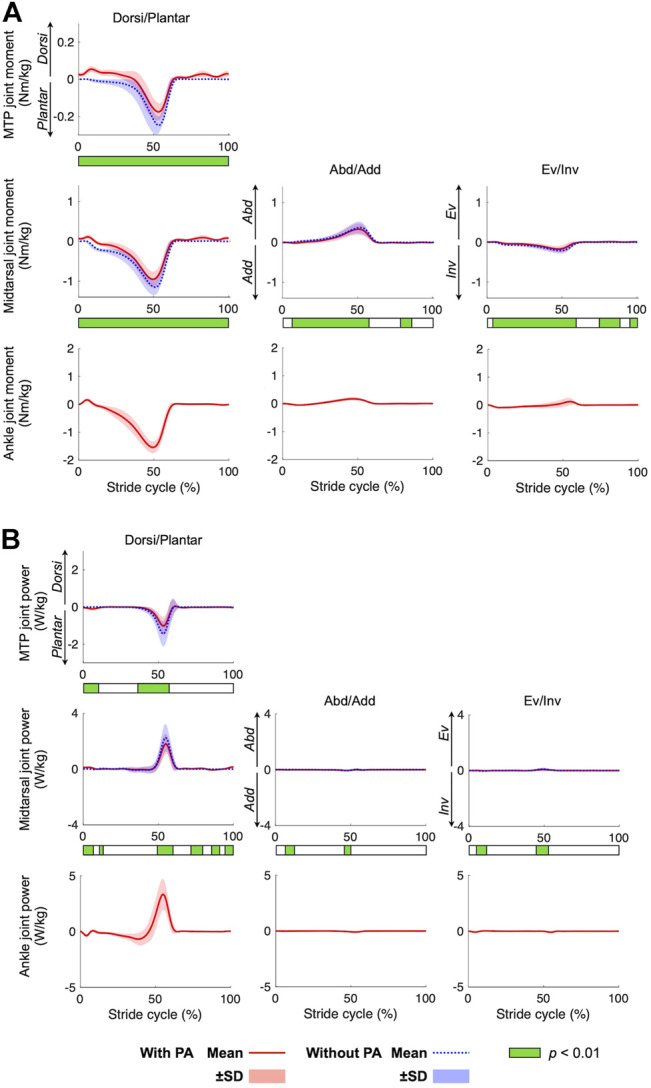
Mean joint moment **(A)** and joint power **(B)** profiles during walking. Mean (solid red line = with PA, dotted blue line = without PA) ± standard deviation (red and blue bands, respectively). Color bar below each graph shows the results of the SPM analysis.

The joint moment and joint power profiles calculated with and without incorporating the PA are presented in Figure 5. Plantarflexion moments were generated by all three joints during walking, particularly in the late stance phase. The magnitudes of the peak plantarflexion moments were 0.18 ± 0.04 Nm/kg, 0.98 ± 0.18 Nm/kg, and 1.58 ± 0.18 Nm/kg for the MTP, midtarsal, and ankle joints, respectively. Slight inversion and abduction moments were generated in phase with the plantarflexion moment by the midtarsal joint. The power generated by the midtarsal joint was calculated to be positive, as the joint plantarflexed while generating the plantarflexion moment. In contrast, the power generated by the MTP joint was negative because the joint dorsiflexed while generating the plantarflexion moment. Therefore, positive and negative work was performed by the midtarsal and MTP joints, respectively, during walking. The peak positive power generated by the midtarsal joint and peak negative power absorbed by the MTP joint were estimated as 1.81 ± 0.73 W/kg and −1.01 ± 0.48 W/kg, respectively, during walking. The power in the eversion−inversion and abduction−adduction directions was almost zero.

**FIGURE 6:**
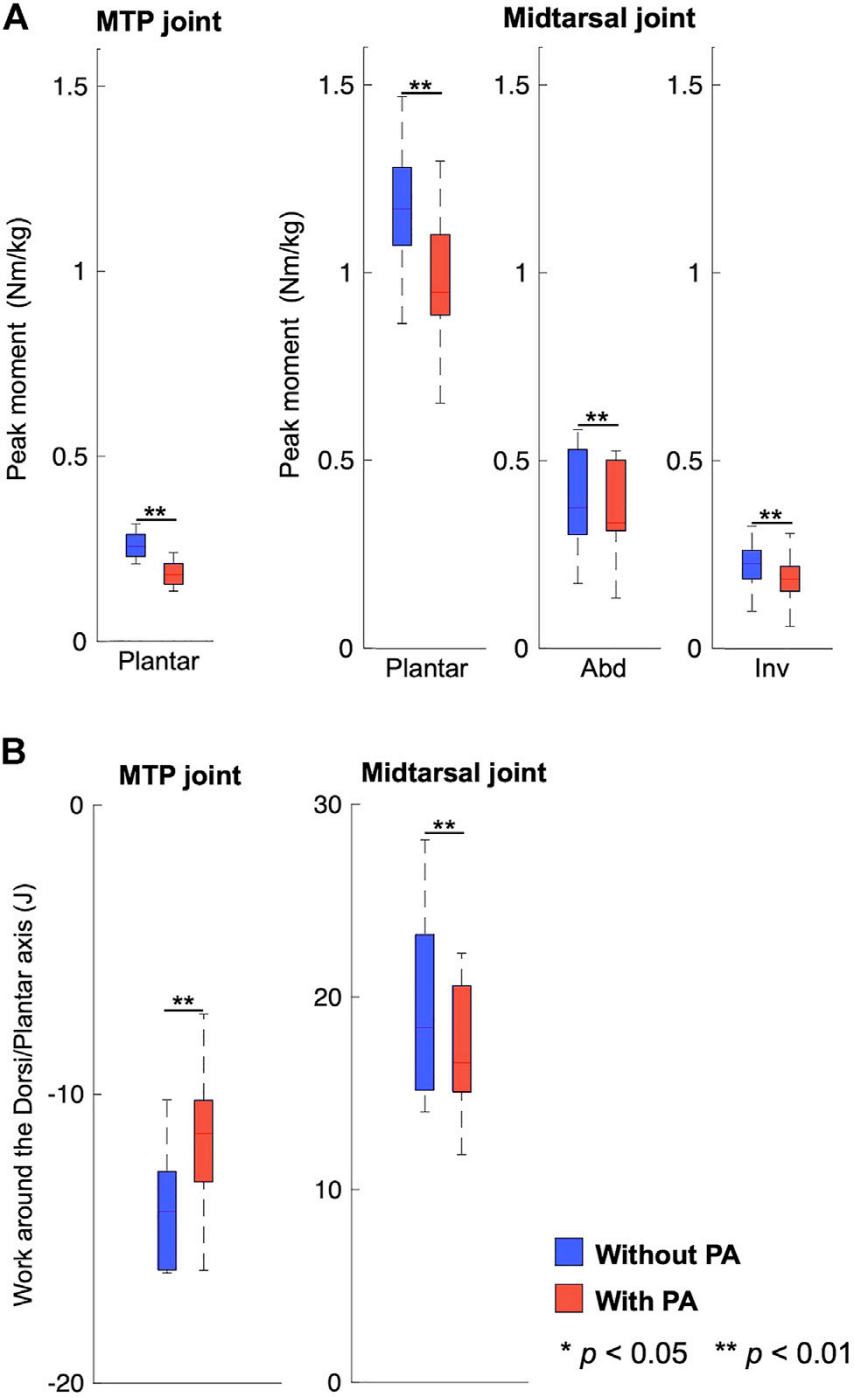
Comparison of the joint moment **(A)** and joint power **(B)** profiles between models with (blue) and without the plantar aponeurosis (red).

Due to the presence of the PA model, the plantarflexion moments and power of the MTP and midtarsal joints were significantly smaller in magnitude in the model with incorporating the PA than that without incorporating the PA (*p* < 0.01). The abduction and inversion moments of the midtarsal joint during the stance phase were also significantly smaller in the model with incorporating the PA than that without incorporating the PA (*p* < 0.01). The mean peak plantar flexion moments of the MTP were 0.07 Nm/kg smaller (*p* = 0.01), and the mean peak inversion, plantar flexion, and abduction moments of the midtarsal joint were 0.02 Nm/kg, 0.2 Nm/kg, and 0.04 Nm/kg smaller, respectively (*p* < 0.01, *p* < 0.01, and *p* = 0.01, respectively) when incorporating the PA model (Figure 6A). The mean negative work done by the MTP joint and the positive work done by the midtarsal joint were significantly reduced by 3.0 and 2.2 J, respectively (*p* < 0.01 and *p* < 0.01, respectively) (Figure 6B).

### 3.4 Sensitivity Analysis

**FIGURE 7:**
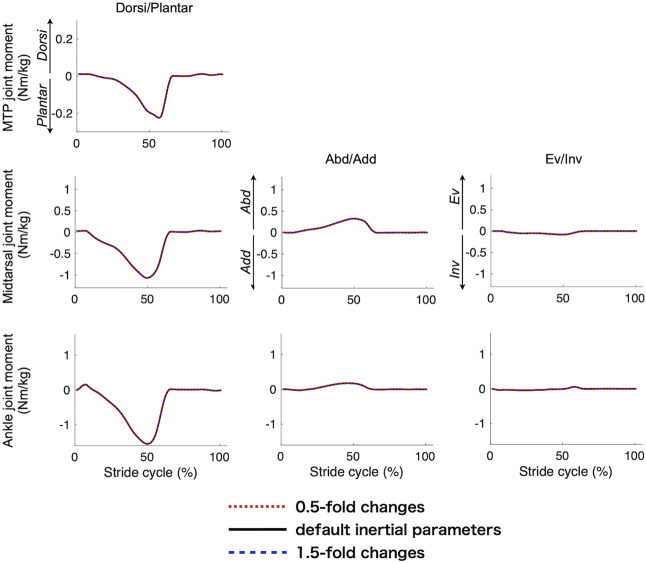
Comparisons of the mean joint moment profiles when the inertial parameters were altered.

**FIGURE 8:**
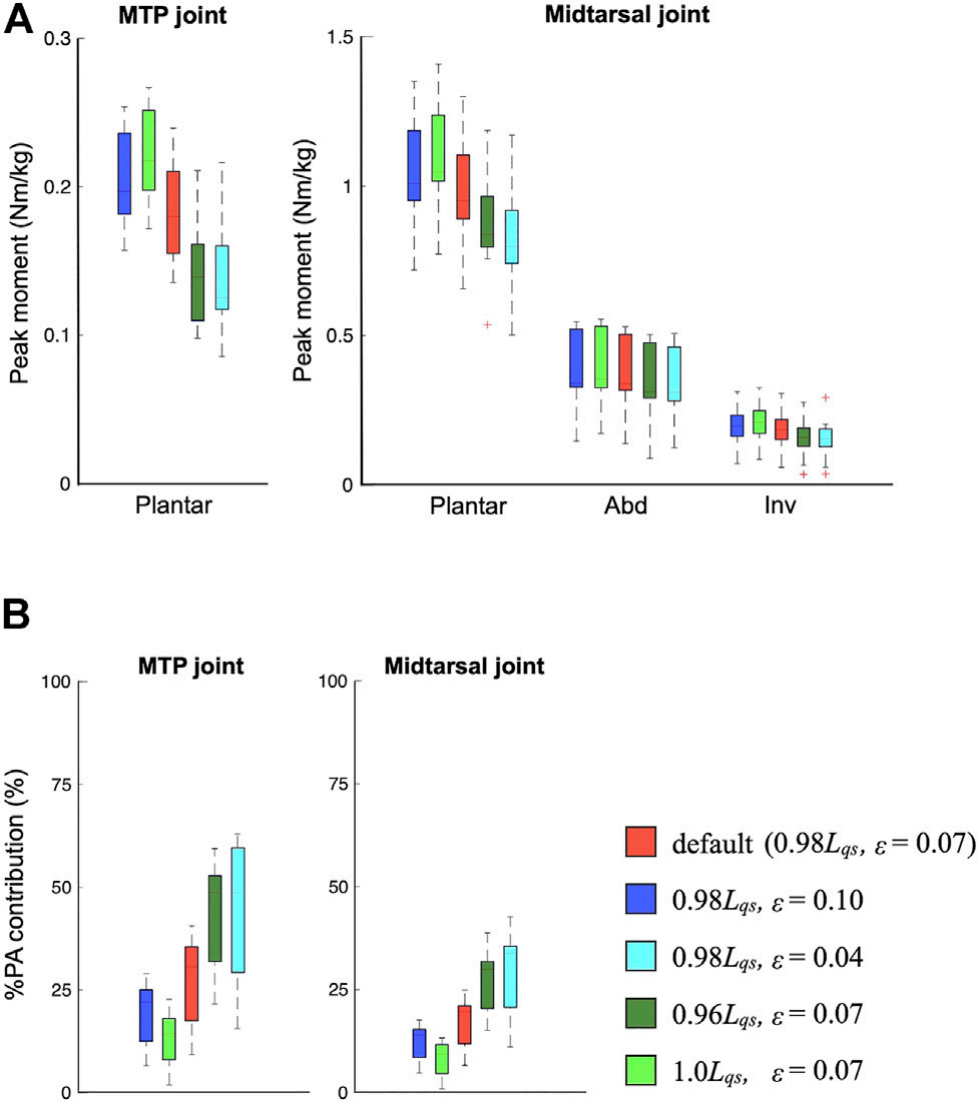
Comparisons of the mean peak moments **(A)** and %plantar aponeurosis (PA) contribution **(B)** at toe-off when the PA stiffness parameters were altered.

Figure 7 compares the mean joint moment profiles of the three joints when the inertial parameters were altered. The change in the inertial parameters had virtually no effect on the calculated joint moments. Figure 8A compares the mean peak moments at toe-off of the midtarsal and MTP joints when the PA stiffness parameters were altered. The change in the PA stiffness altered the peak plantarflexion moments of the two joints but not the moments in the other directions. It was estimated that 13%–45% of the plantarflexion moment of the MTP joint and 8%–29% of plantarflexion in the midtarsal joints were generated by the PA at the time of push-off during walking (Figure 8B).

## 4 Discussion

In this study, we developed a novel multi-segment foot model incorporating the PA for a detailed inverse dynamic analysis of the foot segments. We then ran simulations with the model to address the biomechanical consequences of incorporating the PA on the estimated joint moments and power during human walking. We observed that ∼13%–45% of the plantarflexion moment in the MTP joint and 8%–29% of the plantarflexion moment in the midtarsal joints were generated by the PA at the time of push-off during walking (Figure 8). If the PA was not incorporated in the moment estimation, the joint moments generated by the foot muscles would be overestimated by these amounts. Therefore, we were able to perform a more precise estimation of the joint moments generated within the foot segments, which are not measurable non-invasively, during movements such as walking, running, and jumping. As such, our multi-segment foot model incorporating the PA contributes toward elucidating the basic biomechanics and motor control of the human foot, and to clarify the pathogenic mechanism and possible surgical or rehabilitative interventions for the treatment and prevention of foot pathologies.

We predicted that the PA was stretched in the early and mid-stance phase but shortened in the late stance phase (Figure 4). This is consistent with previous studies reporting the PA elongation profile during walking (Caravaggi et al., 2009, 2010; Welte et al., 2021). However, this study also demonstrated that the midtarsal joint generated positive mechanical work and that the MTP joint generated negative mechanical work in the second half of the stance phase during walking (Figure 5), and if the PA was incorporated in the inverse dynamic calculation, the positive and negative works done by the two joints were both reduced (Figure 6). This might be because the PA springs contributed toward transfer of the energy absorbed at the MTP joint to generate positive work at the midtarsal joint during walking. The human foot possesses a longitudinal arch with the PA spanning its plantar side, allowing stretch and recoil of the PA springs to store and release mechanical energy for generation of efficient locomotion (Ker et al., 1987; Kim and Voloshin, 1995; Stearne et al., 2016). Our study, which was based on inverse dynamics using the proposed multi-segment foot model, clarified the detailed energy recovery mechanism embedded in the human foot that possibly contributes to the reduced energy cost in human bipedal walking.

In the present study, the spring constant of the PA before the split was calculated as approximately 80 N/mm by substituting the mean natural length (171 mm) and mean body weight (63 kg) into Eq. 3, derived based on the published reports that the strain of the PA was approximately 0.07 and the force generated by the PA was approximately 1.5 times the body weight at the time of push-off during walking (see Materials and Methods). The stiffness value is less than a half of the PA stiffness previously obtained *in vitro* (204 N/mm; Kitaoka et al., 1994) and *in vivo* (170 N/mm; Gefen, 2003). However, if the PA is elongated by maximum 6 % and 12% as reported in Caravaggi et al. (2009) and Gefen (2003) during walking, the estimated tensile force generated by the PA will be 2093 and 4186 N, respectively, using 204 N/mm, and 1744 and 3488 N, respectively, using 170 N/mm, presumably too large for the estimated tensile force generated by the PA during walking. Therefore, we believe that the presently estimated stiffness of the PA is of reasonable accuracy, but this must be confirmed by further investigations.

Our study provided inertial parameters of the phalanx, forefoot, and hind foot segments based on the CT scan data of the foot, and we observed that the errors in the inertial parameters had virtually no impact on the joint moment calculation because the inertial forces and moments were much smaller than the others. Thus, the present dataset should serve as a useful reference for inertial parameters of the kinetic multi-segment foot model.

One limitation of the present study was that the kinematic measurements were possibly affected by skin marker artifacts (Nester et al., 2007; Shultz et al., 2011; Schallig et al., 2021). However, skin motion at the foot is generally regarded as relatively small compared to those of other parts of the body (Leardini et al., 2005). Therefore, this limitation should not have a major effect on the current results. Another limitation of our study was that we could not quantitatively validate the estimated PA lengths and forces against the corresponding measured data since *in vivo* direct measurement of the strain and tension generated by the PA during walking is technically impossible. Due to this limitation, we tried to estimate the PA parameters as precisely as possible based on available information and then conducted a sensitivity analysis to show how the uncertainty in the parameters of the PA contributes to the estimated PA forces and joint moments. For more precise estimation of the PA forces and joint moments, efforts should be made to better identify the parameters necessary to quantify the stiffness of the PA based on dissections of cadaver specimens (Guo et al., 2018; Sichting et al., 2020) or medical imaging of living persons using magnetic resonance imaging (Ehrmann et al., 2014; Shiotani et al., 2021), ultrasound imaging (Crofts et al., 2014; Boussouar et al., 2017), and shear wave elastography (Chino et al., 2019; Wang et al., 2019). A third limitation of the study is that the PA model was assumed to be identical for all participants, despite potentially large individual differences in the properties of the PA due to differences in age (Cheng et al., 2014), sex (Pascual Huerta and Alarcón García, 2007; Shiotani et al., 2019), and deformity (Angin et al., 2014, 2018). Such individual differences should also be incorporated in the estimation of the PA forces and joint moments in future studies. A fourth limitation of the study is that the joints of the present foot model were actuated by joint moments instead of muscle forces. Therefore, the model only allows the estimation of the joint moments representing the net effect of the muscles around each joint, but not the estimation of the force generated by each muscle during movements. For this, modeling of the paths of muscles in the foot is necessary, and this should also be investigated in future studies.

## 5 Conclusion

The present study proposed a novel multi-segment foot model incorporating the PA to analyze foot kinetics during dynamic movements and demonstrated the efficacy of the developed foot model by applying it to gait analysis. The present model incorporating the PA predicted that 13%–45% of plantarflexion in the MTP joint and 8%–29% of plantarflexion in the midtarsal joints were generated by the PA at the time of push-off during walking. The present model also demonstrated that the midtarsal joint generated positive work and that the MTP joint generated negative work in the late stance phase. The positive and negative work done by the two joints were both reduced, indicating that the PA contributed towards transfer of the energy absorbed at the MTP joint to generate positive work at the midtarsal joint during walking. The proposed novel foot model may serve as a useful tool to clarify the function and mechanical effects of the PA and the foot during dynamic movements.

## Data Availability

The original contributions presented in the study are included in the article/Supplementary Material, further inquiries can be directed to the corresponding author.
