# Supplementary material for: Novel Multi-Segment Foot Model Incorporating Plantar Aponeurosis for Detailed Kinematic and Kinetic Analyses of the Foot With Application to Gait Studies
Source: Front Bioeng Biotechnol. 2022 Jun 24;10:894731. doi: 10.3389/fbioe.2022.894731 (PMC9265906; doi:10.3389/fbioe.2022.894731)
Supplement: Supplementary file 1 [file Table1.PDF]

frontiers

Supplementary Table S1. Definition of body marker placement.

Number	Description
#1	Front of head, above the temple
#2	Jugular notch where the clavicles meet the sternum
#3	Xiphoid process of the sternum
#4	Acromio-calvicular joint
#5	Lower (right) or upper (left) arm between the elbow (#18) and shoulder (#4) markers
#6	Radial styloid
#7	Ulnar styloid
#8	Anterior superior iliac spine
#9	Lateral upper (right) or lower (left) 1/3 surface of the thigh
#10	Lateral epicondyle of the knee
#11	Head of fibula
#12	Tibial tuberosity
#13	Lateral upper (right) or lower (left) 1/3 surface of the shank
#14	Back of head, roughly in a horizontal plane of front head (#1) markers
#15	Spinous process of the 7th cervical vertebra
#16	Spinous process of the 10th thoracic vertebra
#17	Middle of the right scapula
#18	Lateral epicondyle approximating elbow joint
#19	Upper (right) or lower (left) forearm between the elbow (#18) and finger (#20) markers
#20	Dorsum aspect of the head of the second metacarpal
#21	Posterior superior iliac spine
