# Supplementary material for: Novel Multi-Segment Foot Model Incorporating Plantar Aponeurosis for Detailed Kinematic and Kinetic Analyses of the Foot With Application to Gait Studies
Source: Front Bioeng Biotechnol. 2022 Jun 24;10:894731. doi: 10.3389/fbioe.2022.894731 (PMC9265906; doi:10.3389/fbioe.2022.894731)
Supplement: Supplementary file 2 [file Image1.PDF]

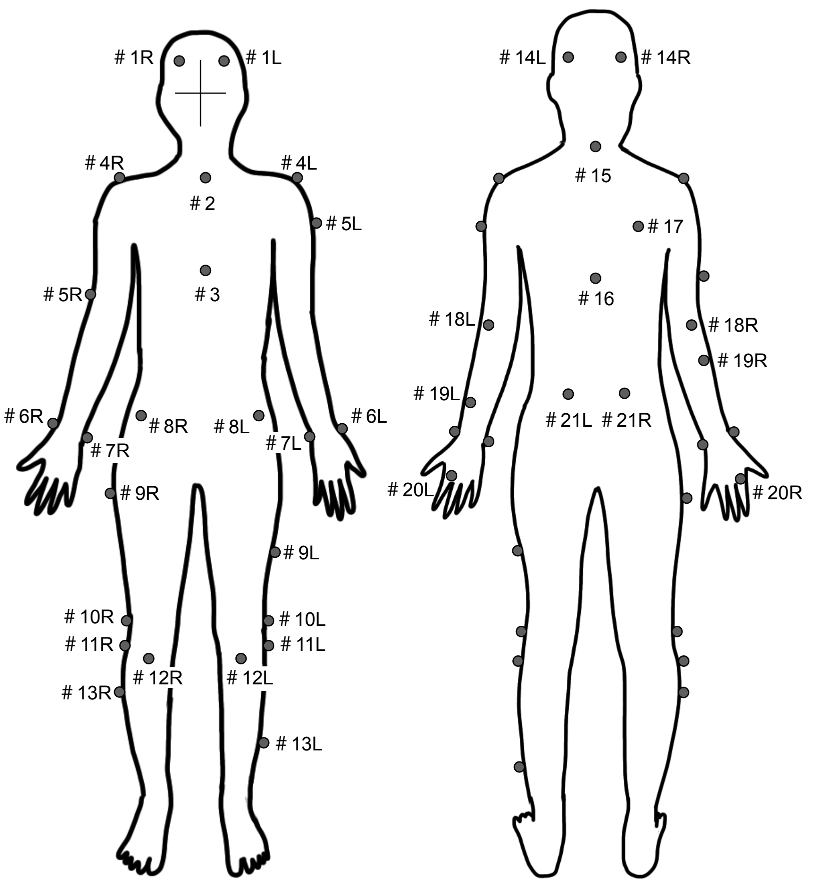

Supplementary Figure S1. Marker placement on the human body.

A

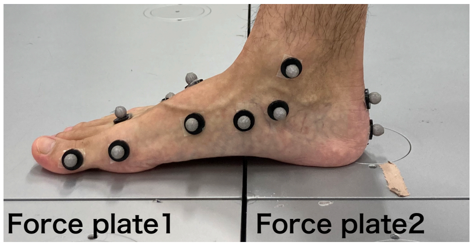

B

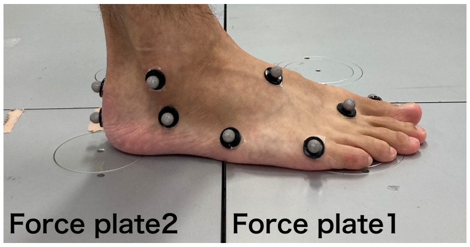

Supplementary Figure S2. A trial with the fore- and hindfoot contacting the front and rear force plates, respectively. This trial was selected for the inverse dynamic analysis. Medial **(A)** and lateral **(B)** views.
